# Transcriptomic Characterization of Tuberculous Sputum Reveals a Host Warburg Effect and Microbial Cholesterol Catabolism

**DOI:** 10.1128/mBio.01766-21

**Published:** 2021-12-07

**Authors:** Rachel P. J. Lai, Teresa Cortes, Suzaan Marais, Neesha Rockwood, Melissa L. Burke, Acely Garza-Garcia, Stuart Horswell, Abdul K. Sesay, Anne O’Garra, Douglas B. Young, Robert J. Wilkinson

**Affiliations:** a The Francis Crick Institute, London, United Kingdom; b Wellcome Centre for Infectious Diseases Research in Africa, Institute of Infectious Disease and Molecular Medicine and Department of Medicine, University of Cape Town, Cape Town, Republic of South Africa; c Department of Infectious Disease, Imperial College London, London, United Kingdom; d MRC Centre for Molecular Bacteriology and Infection, Imperial College London, London, United Kingdom; e Department of Infection Biology, Faculty of Infectious and Tropical Diseases, London School of Hygiene and Tropical Medicine, London, United Kingdom; f National Heart and Lung Institute, Imperial College London, London, United Kingdom; Harvard School of Public Health

**Keywords:** host-pathogen interaction, RNA-Seq, *Mycobacterium tuberculosis*, Warburg effect, cholesterol, cholesterol signaling

## Abstract

The crucial transmission phase of tuberculosis (TB) relies on infectious sputum and yet cannot easily be modeled. We applied one-step RNA sequencing (RNA-Seq) to sputum from infectious TB patients to investigate the host and microbial environments underlying transmission of Mycobacterium tuberculosis. In such TB sputa, compared to non-TB controls, transcriptional upregulation of inflammatory responses, including an interferon-driven proinflammatory response and a metabolic shift toward glycolysis, was observed in the host. Among all bacterial sequences in the sputum, approximately 1.5% originated from *M. tuberculosis*, and its transcript abundance was lower in HIV-1-coinfected patients. Commensal bacterial abundance was reduced in the presence of *M. tuberculosis* infection. Direct alignment to the genomes of the predominant microbiota species also reveals differential adaptation, whereby firmicutes (e.g., streptococci) displayed a nonreplicating phenotype with reduced transcription of ribosomal proteins and reduced activities of ATP synthases, while *Neisseria* and *Prevotella* spp. were less affected. The transcriptome of sputum *M. tuberculosis* more closely resembled aerobic replication and shared similarity in carbon metabolism to *in vitro* and *in vivo* models with significant upregulation of genes associated with cholesterol metabolism and downstream propionate detoxification pathways. In addition, and counter to previous reports on intracellular *M. tuberculosis* infection *in vitro*, *M. tuberculosis* in sputum was zinc, but not iron, deprived, and the *phoP* loci were also significantly downregulated, suggesting that the pathogen is likely extracellular in location.

## INTRODUCTION

Concerted efforts over the last 2 decades have widened availability of therapy for tuberculosis (TB). While this has saved millions of lives, the incidence of disease has declined by only 1.5% annually (1). The host-pathogen interaction in TB is complex, thus hindering the development of diagnostic tests and effective new treatments. Studies on TB rely heavily on *in vitro* or *in vivo* experimental models or blood from TB patients, since lung sampling is invasive. Although these approaches provide insights into TB immune responses and the development of tuberculous lesions at a cellular and molecular level, the events following bacterial release from liquefied lung cavities into the airways remain poorly understood.

Since TB is spread by aerosol generated mainly through coughing, understanding the physiological state of Mycobacterium tuberculosis and its interaction with the host in the nasopharyngeal environment may bring insights on new treatment or preventive therapy strategies. Sputum is routinely collected for TB diagnosis and has been proposed as a surrogate for bronchoalveolar lavage for monitoring the transcriptional profiles of *M. tuberculosis* in patients (2). While several studies in the past have characterized the transcriptomes of sputum *M. tuberculosis* using microarray and/or targeted quantitative PCR, these analyses lacked simultaneous profiling of the host response. We reasoned that a comprehensive RNA sequence-based analysis that yields dual host-pathogen transcriptomes would provide important insight to improve understanding of the biology of *M. tuberculosis* transmission and pathogenesis. Technical difficulties and the overwhelming eukaryotic content have limited conventional sequencing approaches either to the host or to a pathogen that has been physically separated or independently enriched, but dual RNA sequencing (RNA-Seq) allows comprehensive and simultaneous survey of gene expression of both the host and the pathogen in one step. To date, there has been increasing success in using dual RNA-Seq where the technology was successfully applied to profile gene expression of Salmonella enterica in infected HeLa cells (3), Haemophilus influenzae-colonized primary mucosal epithelium (4), and murine Peyer’s patch cells infected with Yersinia pseudotuberculosis (5). Non-one-step dual RNA-Seq has also been used to study Mycobacterium paratuberculosis and Mycobacterium bovis bacillus Calmette-Guerin (BCG)-infected cells *in vitro* but with limited success despite separate microbial enrichment (6, 7). Most recently, dual RNA-Seq studies using *M. tuberculosis*-infected mice have indicated that alveolar and interstitial macrophages utilized different mechanisms to sustain or restrict intracellular *M. tuberculosis* growth (8). In the present study, we applied one-step dual RNA-Seq to sputa collected directly from patients with or without active TB to survey the global transcription profiles of the host and *M. tuberculosis*. The transcriptional signature of the TB-infected host displayed was characteristic of the Warburg effect, while cholesterol catabolism and zinc deprivation were identified in sputum *M. tuberculosis*.

## RESULTS

### Dual RNA-Seq and the host transcriptome.

**FIG 1:**
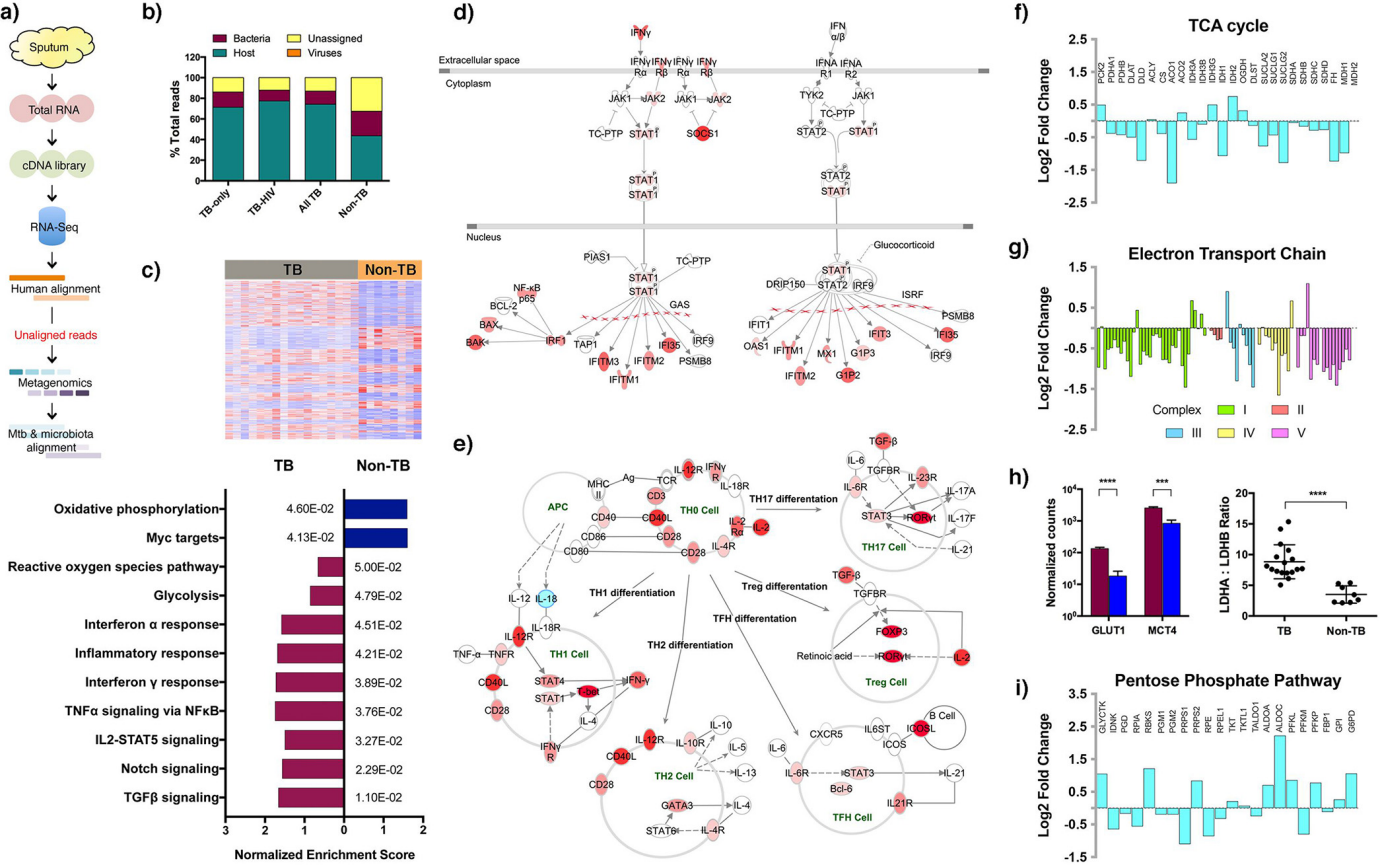
Dual host-pathogen RNA-Seq and the host transcriptome. (a) Sputum samples were collected from 17 active TB and 9 non-TB respiratory symptomatic patients. Total RNA was extracted, and a cDNA library was generated for ultradeep RNA sequencing. Sequence reads were first aligned to the human genome, and unmapped reads were extracted for further microbiome metagenomics classification. After the predominant microbiome taxa were identified, reference-based alignment was performed to the top 10 abundant microbiome species, as well as to *M. tuberculosis*. (b) Global transcript composition profiles of TB and non-TB sputa were calculated. A reduced percentage of host reads and increased percentage of bacterial reads was recorded in non-TB samples. (c) Heatmap showing a total of 5,843 differentially expressed genes in the host transcriptomes between TB (*n* = 17) and non-TB (*n* = 8) sputa. Gene set enrichment analysis identified nine pathways that were significantly enriched in TB and two in non-TB. The *P* value of each enriched pathway is listed. (d) Genes associated with IFN-γ and IFN-α/β signaling pathways were significantly enriched in TB samples. Red indicates upregulation in TB sputa compared to non-TB samples. (e) Evidence of T-cell subset differentiation or recruitment was also observed at the transcriptional level, albeit with generally low read counts. Red indicates upregulation, and blue downregulation in TB versus non-TB sputa. (f and g) Metabolic reprogramming was observed in TB sputa, with decreased expression of genes in the TCA cycle and electron transport chain. The log_2_ fold change of TB sputa compared to non-TB is shown here and is indicative of metabolic reprogramming with a significant decrease in genes involved in the TCA and electron transport chain. The statistical significance of each gene is listed in Data Set S3. (h) In contrast to decreased oxidative phosphorylation, there was a significant increase in genes associated with glucose uptake and lactate export in TB sputa (red) compared to non-TB controls (blue). An increased LDHA/LDHB ratio is indicative of conversion of pyruvate to lactate. Statistical significance (*P* values) is indicated by asterisks (***, *P*-adjusted [*P*-adj] < 0.001; ****, *P*-adj < 0.0001). (i) Transcript expression of genes involved in the NADPH production in the pentose phosphate pathway was also significantly higher in TB sputa. A detailed pathway map with the fold change of significant genes is shown in Fig. S3.

RNA was extracted from 17 sputum samples from South African patients with untreated active TB (9 HIV-uninfected and 8 HIV-infected, referred to as TB-only and TB-HIV, respectively) and 9 samples from persons with respiratory symptoms but no evidence of active TB (referred to as non-TB) (see Data Set S1 in the supplemental material). No physical separation or microbial enrichment was performed to avoid technical error or bias. An average of 1.7 × 10^8^ reads was generated per sample. Sequence reads were first quality filtered and then aligned to the human genome, with unaligned reads extracted for microbiome taxonomy classification and species mapping (Fig. 1a). Regardless of HIV-1 status, human reads accounted for an average of 74% ± 17%, and bacteria accounted for an average of 13% ± 13% of all sequenced reads in tuberculous samples (Fig. 1b). In contrast, non-TB sputa generated significantly fewer human reads (44% ± 20%, *P = *0.0007) and a non-statistically significant higher number of bacterial reads (24% ± 21%). Unassigned reads may have arisen from incomplete filtering of human sequences and from fungal and unidentified bacterial genomes missing from the database.

10.1128/mBio.01766-21.1 DATA SET S1 Patient characteristics. Download Data Set S1, XLSX file, 0.01 MB. Copyright © 2021 Lai et al. 2021 Lai et al. https://creativecommons.org/licenses/by/4.0/ This content is distributed under the terms of the Creative Commons Attribution 4.0 International license.

10.1128/mBio.01766-21.10 FIG S3 Pentose phosphate pathway in the host. The pentose phosphate pathway is illustrated here. The steps in the light green shade represent the oxidative branch of the pathway involved in NADPH production. The transcript abundance of enzymes that mediate the oxidative steps was significantly higher in TB sputa compared to non-TB sputa. In contrast, there was no change or reduction in gene expression associated with the nonoxidative branch of the pathway (shaded in dark green). Download FIG S3, PDF file, 0.1 MB. Copyright © 2021 Lai et al. 2021 Lai et al. https://creativecommons.org/licenses/by/4.0/ This content is distributed under the terms of the Creative Commons Attribution 4.0 International license.

10.1128/mBio.01766-21.3 DATA SET S3 Differentially expressed genes in the human host between TB and non-TB sputa. Download Data Set S3, XLSX file, 1.6 MB. Copyright © 2021 Lai et al. 2021 Lai et al. https://creativecommons.org/licenses/by/4.0/ This content is distributed under the terms of the Creative Commons Attribution 4.0 International license.

We first examined the impact of *M. tuberculosis* and HIV-1 infections on the host transcriptome. We identified 21 genes that, compared to HIV-1-uninfected patients, were differentially expressed in HIV-1-coinfected TB sputa (see Data Set S2), including upregulation of T-cell markers such as CD8A/B, LAG3, and CRTAM. This observation was consistent with that from nonhuman primates with TB, in which coinfection with simian immunodeficiency virus significantly induced LAG3 expression (9), suggesting that T-cell recruitment to TB sputum is quantitatively and qualitatively affected by HIV-1 coinfection. The presence of *M. tuberculosis* had a significant impact on the host transcriptome in the respiratory tract, with total segregation between TB and non-TB samples, in principal component analysis (see Fig. S1). One of the non-TB samples (SP321) was a conspicuous outlier and was omitted from further analysis. Comparison between TB sputa (regardless of HIV-1 status) and non-TB controls identified 5843 genes that were differentially expressed (log_2_ fold change > ±0.5, *P*-adjusted <* *0.05; see Data Set S3). Gene set enrichment analysis of these 5,843 genes identified 11 significant gene sets, 9 of which were positively enriched in TB sputum and 2 of which were negatively enriched in non-TB (Fig. 1c).

10.1128/mBio.01766-21.2 DATA SET S2 Differentially expressed genes in the human host between TB-HIV sputa and TB-only sputa. Download Data Set S2, XLSX file, 1.5 MB. Copyright © 2021 Lai et al. 2021 Lai et al. https://creativecommons.org/licenses/by/4.0/ This content is distributed under the terms of the Creative Commons Attribution 4.0 International license.

10.1128/mBio.01766-21.8 FIG S1 Principal component analysis of host transcript profiles. The host transcriptomes of sputum samples were analyzed by principal component analysis. A complete segregation of the TB (red) from the non-TB (black) samples was observed. One of the non-TB sputa (SP321) was an outlier with differential clustering pattern and was excluded from downstream analysis of the host gene expression. Download FIG S1, PDF file, 0.08 MB. Copyright © 2021 Lai et al. 2021 Lai et al. https://creativecommons.org/licenses/by/4.0/ This content is distributed under the terms of the Creative Commons Attribution 4.0 International license.

The TB enriched pathways consisted of inflammatory responses mediated by interferon gamma (IFN-γ), tumor necrosis factor alpha (TNF-α), and, to a lesser extent, by type I interferon (IFN-α/β) (Fig. 1d). The enhanced transcription of these inflammatory mediators is consistent with elevated cytokine concentrations previously reported in TB sputum compared to pneumonia controls (10). Significant transcriptional changes associated with T helper cell activation and differentiation, including T-bet, GATA3, RORγt, and FOXP3 transcriptional regulators, were also detected despite lymphocytes typically accounting for <1% of the total cellular composition in TB sputum (10) (Fig. 1e). Expression of interleukin-18 (IL-18) was significantly downregulated in TB sputum, while its neutralizing binding protein (IL18BP) was significantly upregulated, suggesting that the increased IFN-γ-mediated response may be driven by IL-12 without IL-18 synergy (11, 12). Furthermore, the increased expression of Th17 and the Foxp3^+^ Treg subsets in TB sputa was consistent with significantly enhanced transcription of transforming growth factor beta (TGF-β). The data show that the host transcriptome in sputum shares both similarities and key differences compared to whole blood (13) and reveal a significant and specific antimycobacterial response in the airways not found in non-TB respiratory conditions.

In parallel with the inflammatory response there was a striking change in host central metabolism in TB sputa, with evidence of a switch from oxidative phosphorylation to glycolysis (see Data Set S3). The expression of genes involved in the tricarboxylic acid (TCA) cycle was significantly downregulated (Fig. 1f) and broken after citrate, with reduced transcription of aconitase (ACO1) and elevated transcription of aconitate decarboxylase (ACOD1/IRG1) (14) (see Fig. S2). The electron transport chain (ETC) (Fig. 1g) was also significantly downregulated in TB sputa, including genes encoding NADH dehydrogenase, cytochrome *c* oxidase, ubiquinol-cytochrome *c* reductase, and mitochondrial ATP (F_0_F_1_) synthase (see Data Set S3). In contrast, there was an enhanced expression of glucose transporter GLUT1 (encoded by SLC2A1) and lactate exporter MCT4 (encoded by SLC16A3) (Fig. 1h), along with a significant increase in the ratio of LDHA to LDHB (lactate dehydrogenases A and B) (Fig. 1h) indicative of increased conversion from pyruvate to lactate (15). Increased transcription of genes involved in the oxidative branch of the pentose phosphate pathway was consistent with production of NAPDH in association with generation of reactive oxygen species (ROS) (Fig. 1i; see also Fig. S3), although transcripts associated with alternative NADPH-generating pathways (cytoplasmic malate dehydrogenase [MDH1], malic enzyme [ME1], and isocitrate dehydrogenase [IDH1]) were found at higher abundance in non-TB sputum. Together, these data support the notion that there is an overall reprogramming of host central metabolism during *M. tuberculosis* infection toward increased glycolysis, either as a positive-feedback mechanism to maintain a fully activated immune response (16) or to produce glycolytic intermediates required for cell proliferation as part of antimicrobial defense (17).

10.1128/mBio.01766-21.9 FIG S2 Itaconate biosynthesis in the host. The TCA cycle of the host in TB sputa was similar to the pattern previously described in M1 inflammatory macrophages, broken after citrate, and resulted in increased production of itaconate. The ACO1 enzyme that converts citrate to *cis*-aconitate and isocitrate was significantly downregulated, whereas IRG1 that mediates conversion to itaconate was significantly induced in TB sputa. Download FIG S2, PDF file, 0.08 MB. Copyright © 2021 Lai et al. 2021 Lai et al. https://creativecommons.org/licenses/by/4.0/ This content is distributed under the terms of the Creative Commons Attribution 4.0 International license.

### Microbiome landscape and its adaptation to *M. tuberculosis* infection.

The inflammatory response revealed by direct transcriptional profiling of sputum samples shares key features common to responses to *M. tuberculosis* infection previously documented in cell culture models and infected human and animal tissues. We anticipated that if this transcription profile was translated into a functional antimicrobial response, it may disrupt the ecology of the commensal respiratory microbiota. To test this hypothesis, we compared the overall microbiome taxonomy and the transcriptional profile of dominant commensal bacterial species between TB and non-TB sputum.

**FIG 2:**
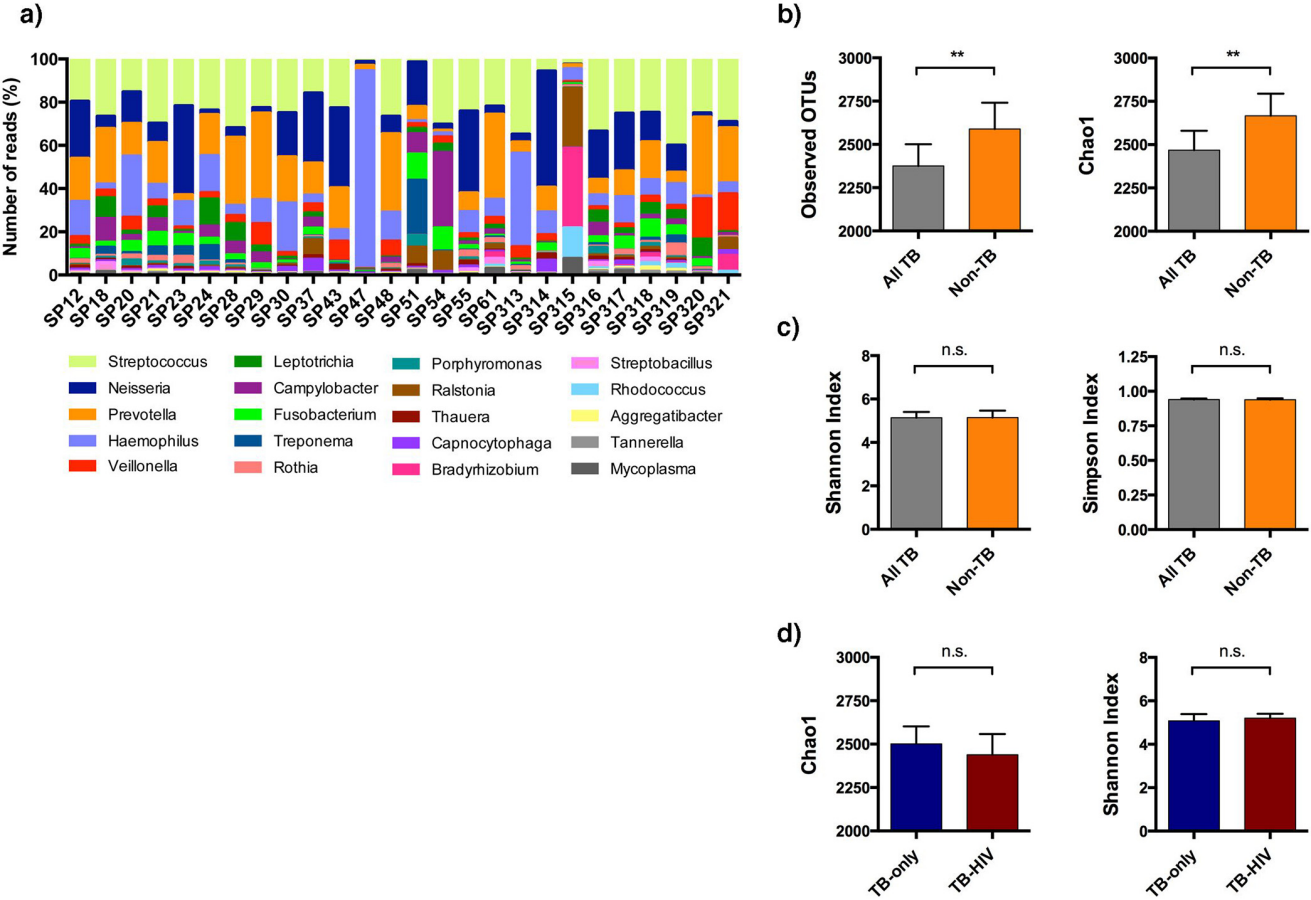
Global overview of sputum microbiome. (a) A stacked bar chart to show the top 20 most represented microbiome genera in TB (SP12-SP61) and non-TB (SP313-SP321) sputa. SP47 had an expansion of Haemophilus and SP315 comprised mainly of known artifacts *Ralstonia* and *Bradyrhizobium*. These two samples were subsequently removed from all downstream analyses. (b) Microbiome species richness and diversity were calculated. Non-TB samples (*n* = 9) had a significantly higher number of observed OTUs and estimated number of true OTUs (chao1 indicator) compared to TB samples (*n* = 17). (c) There was no difference in species diversity, as measured by the Shannon and Simpson indices, indicating species evenness and distribution did not differ between TB and non-TB groups. (d) HIV-1 coinfection did not impact the global microbiome species richness or diversity in sputum. For panels b to d, statistical difference was calculated using Mann-Whitney U-test (*, *P* < 0.05; **, *P* < 0.01; n.s., not significant).

Taxonomic classification of the bacterial reads identified 30 phyla, 613 genera, and 1,331 species (see Data Set S4). Reads mapping to sequenced bacterial genomes ranged from 10^6^ to 10^8^ and the overall taxonomic composition of our TB sputa was similar to that previously reported using 16S DNA (18), with Streptococcus, *Neisseria*, *Prevotella*, Haemophilus, and *Veillonella* being the most represented genera (Fig. 2a). Non-TB sputa had significantly higher microbiome species richness than TB sputa (*P < *0.01 for both operational taxonomic units [OTUs] and Chao1 estimator) (Fig. 2b), but there was no difference in species diversity (Shannon and Simpson indices) (Fig. 2c), indicating that the distribution of species dominance and evenness was not affected by *M. tuberculosis* infection. In keeping with the published literature, similar lung and oral microbiome diversity in HIV-uninfected and HIV-infected patients (19), species richness, or diversity in TB sputa was unaffected by HIV-1 coinfection (Fig. 2d).

10.1128/mBio.01766-21.4 DATA SET S4 Taxonomic classification of sputum microbiome. Download Data Set S4, XLSX file, 0.5 MB. Copyright © 2021 Lai et al. 2021 Lai et al. https://creativecommons.org/licenses/by/4.0/ This content is distributed under the terms of the Creative Commons Attribution 4.0 International license.

### Transcriptional profiling of sputum *M. tuberculosis*.

**FIG 3:**
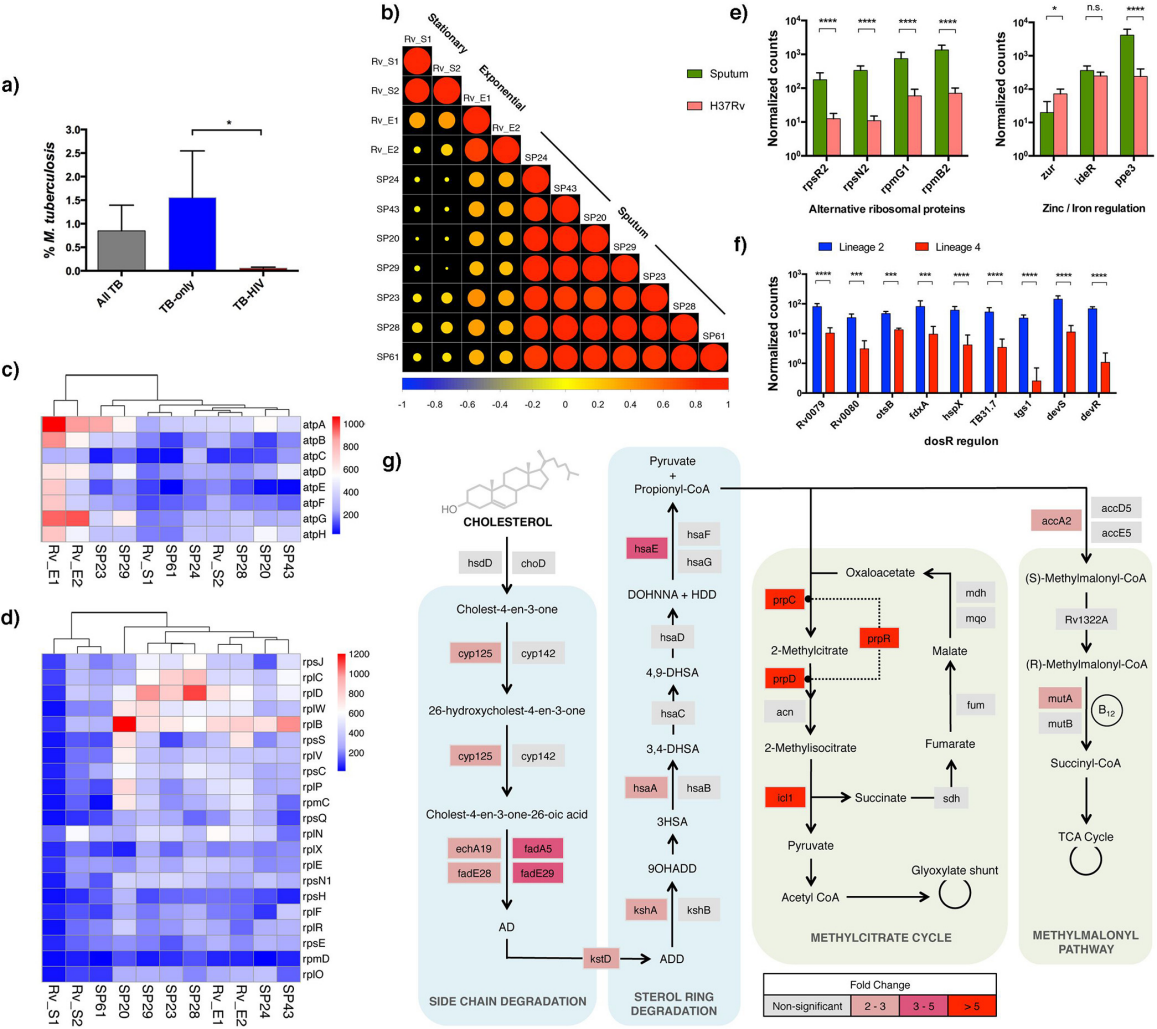
Transcriptional profiles of sputum *M. tuberculosis.* (a) Despite active TB disease, *M. tuberculosis* only accounted for 0.85% ± 2% of all mapped bacterial reads. The percentage of *M. tuberculosis* reads was, however, significantly higher in TB-only samples than in TB-HIV samples (*n* = 9 and *n* = 8, respectively; *P* < 0.05 [Mann-Whitney U-test]). (b) The differential gene expression between seven sputum *M. tuberculosis* samples and laboratory cultures was calculated using DESeq2. The expression data were plotted as a correlation matrix with hierarchical clustering. Exponential cultures were labeled as Rv_E1 and Rv_E2, stationary cultures were labeled as Rv_S1 and Rv_S2, and sputum samples started with the initials SP. A decrease in circle size indicates reduced correlation; red indicates a positive correlation, and blue indicates a negative correlation. The sputum samples showed a high degree of concordance to each other and correlated more closely to exponential-phase cultures than to stationary-phase cultures. (c) Transcript abundance of ATP synthase genes in sputum *M. tuberculosis* clusters more closely to stationary-phase H37Rv than to exponential-phase cultures. (d) In contrast, transcript abundance of the two major ribosomal protein operons S10 and L14 in sputum *M. tuberculosis* were found to be more similar to exponential-phase H37Rv than to stationary-phase cultures. The color of the heatmaps corresponds to the normalized read count of each gene. (e) Significantly higher expression of four zinc-independent alternative ribosomal proteins was detected, along with decreased expression of the *zur* repressor and upregulation of *ppe3*, indicating that sputum *M. tuberculosis* was zinc deprived. (f) Expression of selected members of the DosR regulon. Consistent with the presence of an alternative transcriptional start sites in lineage 2 isolates (22), the transcript abundance of the DosR genes was significantly higher in lineage 2 sputum than in lineage 4 sputum *M. tuberculosis*. (g) Compared to exponential-phase laboratory cultures (H37Rv), *M. tuberculosis* in sputum was found to have significantly higher expression of 34 members of the KstR and KstR2 regulons associated with cholesterol catabolism and 6 members of the downstream propionate detoxification pathways. A pathway map is shown here to illustrate the transcript expression of some of the enzymes involved in the processes. Genes that were not differentially expressed (nonsignificant) are colored in gray, and those that were differentially expressed in sputum were colored in a scale of pink and red colors according to their fold change. No downregulated genes were identified in the KstR/KstR2 regulons or either of the propionate detoxification pathways. For panels e to f, adjusted *P* values (*P*-adj) were determined by DESeq2 and are indicated by asterisks (*, *P*-adj < 0.05; **, *P*-adj < 0.01; ***, *P*-adj < 0.001; ****, *P*-adj < 0.000; n.s., not significant).

Reads mapping to *M. tuberculosis* accounted for only 0.85% ± 2% of total mapped bacterial reads (Fig. 3a), ranging from 10^3^ to 10^5^. Consistent with evidence of lower transmission from HIV-1 coinfected patients (20), there was a significantly higher percentage of *M. tuberculosis* reads in TB-only, compared to the TB-HIV sputa (mean, 1.55% versus 0.06%, respectively; *P = *0.027) (Fig. 3a).

Seven samples (6 TB-only and 1 TB-HIV) had sufficient read coverage (>4 × 10^4^ reads) to quantify transcript abundance for >50% of the *M. tuberculosis* genome. Three of the samples were identified as belonging to lineage 2, one to lineage 3, and three to lineage 4 (see Data Set S1). In the obvious absence of a comparative control from non-TB sputa, we compared the sputum *M. tuberculosis* transcriptome to exponential- and stationary-phase liquid laboratory cultures of *M. tuberculosis* strain H37Rv. Plotting expression data as a correlation matrix demonstrated that the sputum profiles formed a closely related cluster that shared greater similarity to exponential than to stationary-phase culture (Fig. 3b). Expression analysis identified 198 genes as differentially expressed between sputum and exponential culture (*P-*adjusted* *<* *0.05; see Data Set S5), and 392 genes between sputum and stationary phase (*P-*adjusted* *<* *0.05; see Data Set S6).

10.1128/mBio.01766-21.5 DATA SET S5 Differentially expressed genes in sputum *M. tuberculosis* compared to exponential-phase H37Rv. Download Data Set S5, XLSX file, 0.03 MB. Copyright © 2021 Lai et al. 2021 Lai et al. https://creativecommons.org/licenses/by/4.0/ This content is distributed under the terms of the Creative Commons Attribution 4.0 International license.

10.1128/mBio.01766-21.6 DATA SET S6 Differentially expressed genes in sputum *M. tuberculosis* compared to stationary-phase H37Rv. Download Data Set S6, XLSX file, 0.04 MB. Copyright © 2021 Lai et al. 2021 Lai et al. https://creativecommons.org/licenses/by/4.0/ This content is distributed under the terms of the Creative Commons Attribution 4.0 International license.

Transcript abundance across the ATP synthase operon in sputum was closer to stationary-phase than to exponential-phase cultures (Fig. 3c), whereas transcription of the main ribosomal protein operons more closely resembled the exponential reference (Fig. 3d). A striking feature of the ribosomal protein gene profile in sputum was a high abundance of transcripts for a set of four alternative ribosomal proteins characteristic of growth in a low-zinc environment (Fig. 3e). Additional zinc-regulated genes (21), including the putative chaperone Rv0106, methyltransferase Rv2990c, and the ESX-3 operon, were also significantly increased in sputum compared to laboratory culture (see Data Sets S5 and S6). The ESX-3 operon is under dual control of zinc-responsive Zur and iron-responsive IdeR repressors; induction of *ppe3*, which lies upstream of the IdeR site and downstream of a Zur site, provides further indication of zinc deprivation (Fig. 3e). Expression of the DosR stress regulon in sputum more closely resembled the exponential-phase than the stationary-phase reference (see Data Sets S5 and S6), with significantly higher expression of *dosR* genes in sputum samples infected with lineage 2 compared to lineage 4 isolates (Fig. 3f). Inspection of expression profiles showed that this reflected an increase in *dosR* transcripts originating from a single nucleotide polymorphism (SNP)-generated constitutive start site internal to Rv3134c in lineage 2, rather than from the stress-inducible start site upstream of Rv3134c (22–24) (see Fig. S3).

Thirty-four members of the KstR and KstR2 regulons involved in the degradation of the cholesterol side chain and ABCD rings (25) and genes involved in downstream propionate metabolism by the methylcitrate cycle (26) and methylmalonate pathways (27) were consistently higher in sputum than in laboratory culture (Fig. 3g). This is similar to previous descriptions of the induction of *M. tuberculosis* cholesterol catabolism genes in macrophage and mouse models (28, 29). PhoP plays an important role in transcriptional regulation during *M. tuberculosis* infection, and analysis by chromatin immunoprecipitation has identified a set of genes that are regulated by binding of PhoP to upstream sites (30). Twenty PhoP-regulated transcripts, including small RNA *mcr7*, were differentially expressed in sputum compared to laboratory culture; in all but one case, the sputum profile was consistent with a decrease in PhoP binding (see Data Set S5).

We validated 15 differentially expressed genes using NanoString methodology and compared the transcript levels in three sputum samples against an independent *M. tuberculosis* H37Rv reference culture. These included representative upregulated (KstR, Zur, and propionate) and downregulated (ATP and mycobactin synthesis) genes. All genes showed the same pattern of differential expression (see Data Set S7) and validated the use of dual RNA-Seq in studying *M. tuberculosis* transcriptome despite its minor representation among the microbial community.

10.1128/mBio.01766-21.7 DATA SET S7 Validation by NanoString. Download Data Set S7, XLSX file, 0.01 MB. Copyright © 2021 Lai et al. 2021 Lai et al. https://creativecommons.org/licenses/by/4.0/ This content is distributed under the terms of the Creative Commons Attribution 4.0 International license.

## DISCUSSION

M. tuberculosis spends most of its life sequestered in lesions within tissues, but in order to transmit to a new host, it has to move into the respiratory tract prior to release in the form of infectious aerosol droplets (31). The transmission phase is difficult to model in experimental systems and is poorly understood. We reasoned that sputum samples could be exploited to obtain additional information about conditions in the respiratory tract that may influence the efficiency of TB transmission. We generated RNA sequence data directly from sputum and analyzed these with respect to host, pathogen, and microbiome transcripts to provide a comprehensive overview of the entire ecosystem. This is the first report that such a strategy can be successfully applied to pathological specimens, with manifest implications for the study of other human infectious diseases to complement *in vitro* and animal models.

Comparison of host transcript profiles from TB patient sputum with *M. tuberculosis*-negative sputum revealed wholesale changes characteristic of the innate and adaptive immune inflammatory response. Given the unpromising physical appearance of sputum as a heterogeneous mixture of cell debris and mucoid secretions, the homogeneity and clarity of the transcriptional response is striking and may reflect elimination of signal from dead cells by mRNA degradation. As in previous clinical studies using whole blood (32), we detected a strong type I/II interferon-mediated cytokine responses in sputum, but a strong T-cell activation and differentiation signature detected in sputum is not seen in blood, likely reflecting sequestration of these cells at the site of disease. These changes were accompanied by a metabolic shift toward glycolysis with a reduction in oxidative phosphorylation and a broken TCA cycle (33). The Warburg effect in mycobacterial infection is IFN-γ-dependent (34) and probably results from a functional change in the mitochondria from energy generation to production of ROS. Upregulation of superoxide dismutase, myeloperoxidase, and glutathione peroxidase were identified in TB sputa (see Data Set S3), implicating a shift in the role of host mitochondria toward bactericidal activity. A switch to glycolysis, which allows the rapid production of ATP, would therefore compensate for energy loss and maintain the mitochondrial membrane potential, while upholding antimicrobial defense mechanisms.

Although the majority of microbiome studies focus on the intestine, there is increasing interest in respiratory microbiota (35). Only a few studies have examined the microbiome in TB (18, 36, 37). The bacterial species detected by sputum RNA sequencing in our cohort are similar to those reported in other studies of the oral cavity and respiratory tract, reflecting the inevitable mixing associated with coughing and expectoration, and include a combination of aerobic and anaerobic members of firmicute, bacteroidetes, and proteobacterial phyla. As reported in previous studies of the lung microbiome, we did not observe any major impact of HIV-1 infection on taxonomic distribution (19). In a recent 16S rDNA-based analysis of tuberculous and nontuberculous sputa, no association between the sputum microbiota composition and TB disease or variation throughout anti-TB treatment could be found in three different settings (38). The authors suggested transcriptomic approaches may provide greater power and, in this single-center study, we did find a significant reduction in species richness in TB sputum compared to non-TB sputum. Intriguingly, despite having active disease, *M. tuberculosis* only accounted for a very small percentage of total bacterial reads measured and was very small in those with HIV-1.

It is likely the change in pattern of metabolism in tuberculous sputum we describe is majorly contributed to by neutrophils since these cells are the predominant infected phagocytic cells in the airways of patients with active pulmonary TB (39). It is recognized that even minimal tuberculous lesions can be sensitively detected by uptake of the false substrate [^18^F]fluorodeoxyglucose, most likely by neutrophils (40). We did not perform cell counts on sputum, and single-cell RNA sequencing analysis of sputum would likely be highly demanding. Thus, our ability to deconvolute the cellular origin of the host sputum transcriptome is limited. We did detect the simultaneous overrepresentation of type I and II interferon pathways in sputum, recapitulating findings in peripheral blood (13) and more recently found in the lungs of mice in conjunction with increased glycolysis (41). We also acknowledge that the total read counts detected for *M. tuberculosis* are low for typical differential gene expression analysis. This is due to the one-step protocol in which no bacterial enrichment was performed in order to accurately assess the abundance of *M. tuberculosis* in its natural environment and to avoid the induction of transcriptomic changes during the enrichment process. Despite the low read counts and its scarce representation among the total bacterial population, there was an overwhelming upregulation of genes associated with cholesterol catabolism (29, 42). The ability of *M. tuberculosis* to utilize cholesterol is unique among the major species in the respiratory microbiome since *M. tuberculosis* can shunt the toxic byproduct (propionate) into the methylcitrate cycle and the methylmalonyl pathway, which may be of a crucial adaptive significance. The *M. tuberculosis* sputum transcriptome also reveals evidence of zinc deprivation. This is of particular interest in light of evidence that the bacteria face the opposite challenge of zinc intoxication when phagocytosed by activated macrophages (43). Neutrophil-derived calprotectin may restrict the availability of zinc in the respiratory tract, and competition with commensals for free zinc may represent a vulnerability of *M. tuberculosis* in sputum. It has been proposed that zinc limitation defines a population of *M. tuberculosis* with anticipatory adaptations against impending immune attack, based on the evidence that zinc-limited *M. tuberculosis* is more resistant to oxidative stress, exhibits increased survival, and induces more severe pulmonary granulomas in mice (44). Similarly, in contrast to results in macrophage cultures (45), the *M. tuberculosis* sputum transcriptome is characterized by reduced activation of the PhoP regulon compared to exponential-phase cultures. Several studies have partially characterized the transcriptome of *M. tuberculosis* from sputum or bronchoalveolar lavage using whole-genome probed-based qPCR or microarray (2, 46–49). There is significant common ground in energy metabolism, ATP synthesis, iron response, and the PhoP regulon when comparing our data to these studies, but with key differences in the DosR regulon. The expression of DosR genes in sputum *M. tuberculosis* has been described to resemble hypoxic nonreplicating laboratory cultures (47, 49), distinct from both aerobic and hypoxic cultures (2) and found in lower abundance in HIV-1-coinfected patient samples when the lineage was controlled (50). The discrepancies could be due to the geographic location and lineage of the samples collected, sample preparation, the technology used for quantification, and the growth conditions and origin of the laboratory cultures used for comparison. Finally, it will be important to determine the ratio of extracellular to intracellular *M. tuberculosis* in sputum; while there is clearly recruitment of an activated population of inflammatory cells in TB sputum, it is possible that these cells are engaged in phagocytosis of commensal bacteria rather than *M. tuberculosis*.

### Conclusions.

The overall aim of our research was to identify interventions that will reduce the viability of *M. tuberculosis* in the respiratory tract in order to reduce the efficiency of infection and transmission. We anticipate that this could involve (i) vaccination to prime effective T-cell responses and opsonizing antibodies, (ii) targeted antibody or small molecule therapies to optimize host responses, and (iii) nutritional or antibiotic interventions that alter the respiratory microbiome. Comprehensive mapping of the transcriptional landscape of both the host and the *M. tuberculosis* described here provides a crucial framework for further study.

## MATERIALS AND METHODS

### Patient cohort and sample collection.

The study was conducted at the Ubuntu Clinic, an integrated HIV/TB outpatient facility in Khayelitsha Site B, Cape Town. Adult (≥18 years old) patients starting TB treatment for confirmed pulmonary TB, as evidenced by a sputum sample that was (i) smear positive for acid-fast bacilli or (ii) positive for *M. tuberculosis* by Xpert *M. tuberculosis*/Rif (Cepheid) testing, were recruited for the study. Additional sputum samples from respiratory symptomatic non-TB patients were collected subsequently. TB disease was excluded when patients did not meet these two criteria and had no radiographic evidence of TB. Demographic data (age and sex), HIV status, CD4 count, and antiretroviral therapy (ART) prescription (if HIV-1 infected) are recorded in Data Set S1. Spontaneously produced sputum was collected from each patient recruited prior to treatment initiation. Sputum samples were collected in a 40-ml specimen jar and TRIzol reagent (Life Technologies) was added in a 2:1 ratio (i.e., 2 ml of TRIzol to 1 ml of sputum) with a pipette. The specimen jar was then closed and shaken to homogenize the sputum. Samples were stored at −80°C until use.

### Ethical statement.

The Human Research Ethics Committee of the Faculty of Health Sciences of the University of Cape Town approved the study (HREC references 031/2012 and 568/2012) and written informed consent was obtained from all participants. No identifiable individual personal data are included here.

### Bacterial strains and growth conditions.

M. tuberculosis H37Rv (SysteMTb strain) was grown in Middlebrook 7H9 medium (Sigma-Aldrich) with 10% albumin dextrose catalase supplement (Sigma-Aldrich), 0.2% glycerol, and 0.05% Tween 80. Exponential-phase mycobacterial cultures were grown to an optical density at 600 nm (OD_600_) between 0.6 and 0.8 in roller bottle at 37°C and 2 rpm. Stationary-phase cultures were grown for 4 weeks after the OD_600_ reached 1.0. Bacteria were harvested by centrifugation at room temperature for 5 min at 2,000 × *g*. TRIzol reagent was immediately added to the bacterial pellet in a 2:1 ratio, followed by vigorous vortexing for homogenization. Samples were stored at −80°C until use.

### RNA extraction.

TRIzol preserved sputum or H37Rv cultures (2 mL) were thawed immediately before RNA extraction. Samples were ribolyzed (i.e., processed in a Ribolyzer sample homogenizer) twice with 0.1-mm silica spheres (MPBio) with a setting of 6 m/s for 45 s. Ribolyzed samples were immediately placed on ice and centrifuge briefly. Chloroform (200 μl) was added to each milliliter of lysed sample and vortexed for 1 min before centrifugation at 10,000 × *g* for 1 min. The aqueous phase was carefully transferred to a new Eppendorf tube, mixed rigorously with equal volume of chloroform-isoamyl alcohol (24:1), and centrifuged at 10,000 × *g* for 5 min. The aqueous phase was carefully transferred to a new Eppendorf tube and mixed with an equal volume of 100% ethanol. The mixture was then passed through a Zymo-Spin IC column (Zymo Research) where nucleic acids were captured in the membrane. The column was treated twice with 10 U of Turbo DNase (Thermo Fisher Scientific) and 100 U of RNase inhibitor (TaKaRa Clontech) at 37°C for 30 min until DNA-free. The DNase-treated RNA was then purified using the RNA Clean & Concentrator-5 kit (Zymo Research) and eluted in nuclease-free water. RNA was extracted from 26 sputum samples and from 4 culture samples, and their quantity and quality were determined by using a Qubit fluorometer (Thermo Fisher Scientific), a NanoDrop spectrophotometer (Thermo Fisher Scientific), and Caliper LabChip systems (Perkin-Elmer).

### Library preparation and RNA-Seq.

RNA-Seq libraries for the 26 sputum samples and 4 culture samples were prepared with 200 ng of corresponding RNA using the Ovation Human FFPE RNA-Seq multiplex system (NuGen), which includes proprietary oligonucleotides for the removal of human rRNA and customized oligonucleotides to remove the rRNA of *M. tuberculosis*. The cDNA was sheared to ∼200 bp with a Covaris E220 ultrasonicator (Covaris) prior to adaptor ligation and amplification. All cDNA libraries were quantified using a Qubit fluorometer and quality checked using a DNA-1000 kit (Agilent) on a 2100 Bioanalyzer. Each sputum library was loaded onto a single lane in a flow cell and sequenced with a Hi-Seq 2500 instrument (Illumina). With the exception of four samples (Rv_E1, Rv_S1, SP55, and SP61) where only ∼100 million 100-bp single-end reads were obtained, all other sputum samples and laboratory cultures (Rv_E2 and Rv_S2) generated ∼200 million 100-bp single-end reads.

### Read mapping and read counting.

The quality of the Illumina-produced fastq files was assessed using FastQC, and poor-quality reads were trimmed using the SolexaQA package (51) using default parameters, trimming bases with confidence *P *> 0.05 and removing reads of <25 bases. The good-quality reads were mapped to the human genome (NCBI GRCh38 build) using Tophat2 with the default parameters (52). The nonhuman reads were then exported for taxonomic classification using Kraken (see below) and subsequently aligned to reference genomes of *M. tuberculosis* and commensal bacteria (see Data Set S6 for accession numbers and references) as single-end data using BWA v0.7.12 (53), and genome coverage was calculated using BEDTools (54). The lineage of the sputum *M. tuberculosis* was determined using the KvarQ algorithm (55) and scanned with the SNPs testsuite.

### Read count normalization and differential gene expression analysis.

Date were analyzed in R v3.5.2. Read count normalization was performed using DESeq2 (56), which is based on a negative binomial distribution model. DESeq2 also determined the fold change between sputum *M. tuberculosis* and H37Rv cultures or between TB and non-TB samples (results are shown as the log_2_ fold change). Statistical significance was calculated and adjusted using the Benjamini-Hochberg multiple testing method with a false discovery rate of 10% (shown as *P*-adjusted). Differentially expressed genes in *M. tuberculosis* that are statistically significant were used to generate a correlation matrix using corrplot with hierarchical clustering (57). A heatmap was created for the differentially expressed genes in the host transcriptome using gplots. Pathway analysis of differentially expressed genes was performed using Gene Set Enrichment Analysis (58) and IPA Ingenuity (Qiagen) for human data and the KEGG pathway (59) for bacterial data.

### Taxonomic classification of sequenced reads.

For each of the 26 sputum samples (17 samples from patients with untreated active TB and 9 additional samples from patients who were non-TB respiratory symptomatic), the set of nonhuman reads was used for taxonomic classification using Kraken (60) screening against the reference MiniKraken database representing complete bacterial, archaeal, and viral genomes in RefSeq. Classification results were visualized using Krona (61). The percent representation of *M. tuberculosis* was calculated relative to the total bacterial sequences identified, and statistical differences between TB and TB-HIV groups were calculated using the nonparametric Mann-Whitney U-test in Prism 6 software.

### Taxonomic diversity and comparative analysis among sputum samples.

Taxonomic reports derived from Kraken were imported into QIIME (62) for the comparative analysis of microbiome species richness and diversity among TB, TB-HIV, and non-TB samples. Species richness was calculated based on the number of observed operational taxonomic units (OTUs) and the Chao1 estimator, which estimates the real species richness based on OTUs. The species diversity, which indicates evenness and distribution, was estimated using Shannon and Simpson indices. The statistical difference between different sample groups was calculated using a nonparametric Mann-Whitney U-test in Prism 6 software.

### NanoString validation of gene expression.

A set of 15 differentially abundant transcripts identified by RNA-Seq was reinvestigated and validated using a customized NanoString nCounter assay (63) (Codeset ID “M.tuberculosisH37Rv”; NanoString Technologies). An independent set of triplicate H37Rv (exponential-phase culture) was prepared as described above and total RNA extracted. Three sputum samples (SP28, SP29, and SP61) that were previously used for RNA-Seq library construction had sufficient quantity of RNA remaining and were used in the NanoString nCounter assay. Hybridization and scanning using a NanoString assay were performed by the UCL NanoString facility according to the manufacturer’s instructions. Briefly, customized barcoded capture/reporter probe pairs specific for each transcript were hybridized overnight at 65°C to 2.5 μg of total RNA for sputum samples and 5 ng of total RNA for culture samples. Positive- and negative-control probe pairs were also included. Unhybridized probes were removed, and the hybridized probes were purified on an nCounter Prep Station. The barcode on each reporter probe was scanned with an nCounter digital analyzer to generate a quantitative measure of the hybridized RNA. Sample signal values were subtracted for background, defined as the mean number of counts for negative-control probes plus 1 standard deviation. The filtered signal values were then normalized using DESeq2 and differential expression between sputum samples and exponential-phase cultures were computed as described above.

### Data availability.

The RNA-Seq data reported here have been deposited in the European Nucleotide Archive under study number ERP012221 and accession number PRJEB10919. All data generated or analyzed during this study are included in this published article and its supplemental material.
