# Supplementary material for: Transcriptomic Characterization of Tuberculous Sputum Reveals a Host Warburg Effect and Microbial Cholesterol Catabolism
Source: mBio. 2021 Dec 7;12(6):e01766-21. doi: 10.1128/mBio.01766-21 (PMC8649757; doi:10.1128/mBio.01766-21)
Supplement: FIG S3 [file mbio.01766-21-sf003.pdf]

SI Figure S3

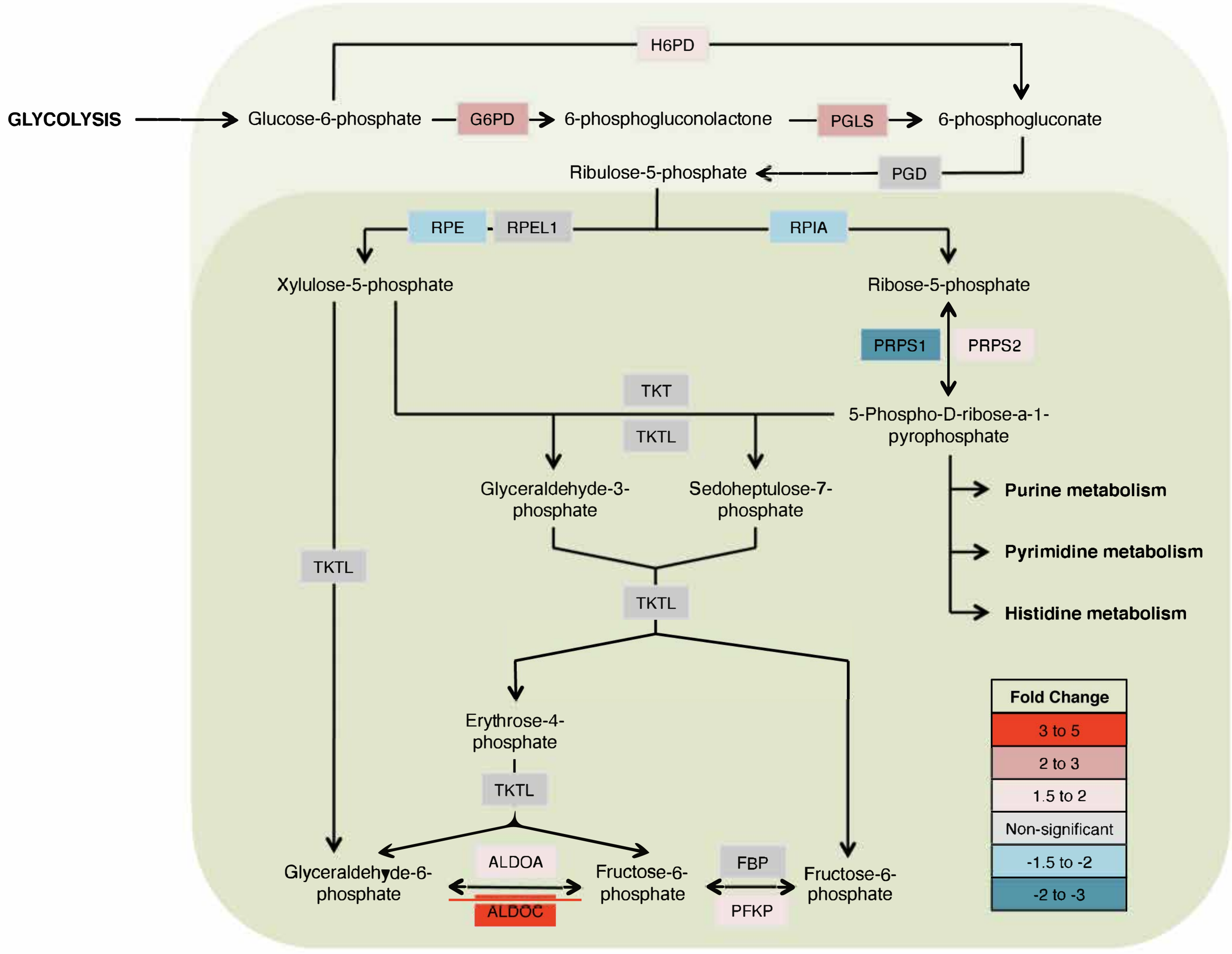

Supplemental Data Figure S3. Pentose phosphate pathway in the host

The pentose phosphate pathway is illustrated here. The steps in the light green shade represent the oxidative branch of the pathway involved in NADPH production. Transcript abundance of enzymes that mediate the oxidative steps was significantly higher in TB sputa compared to non-TB. In contrast, there was no change or reduction of gene expression associated with the non-oxidative branch of the pathway (shaded in dark green).
