# Supplementary material for: Transcriptomic Characterization of Tuberculous Sputum Reveals a Host Warburg Effect and Microbial Cholesterol Catabolism
Source: mBio. 2021 Dec 7;12(6):e01766-21. doi: 10.1128/mBio.01766-21 (PMC8649757; doi:10.1128/mBio.01766-21)
Supplement: FIG S2 [file mbio.01766-21-sf002.pdf]

SI Figure S2

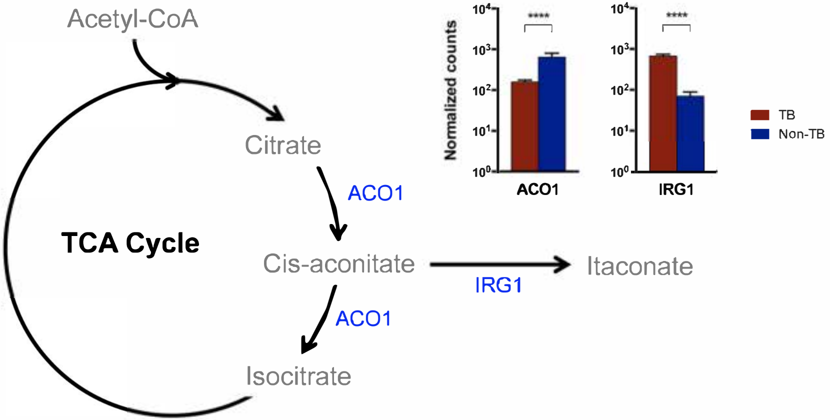

Supplemental Data Figure S2. Itaconate biosynthesis in the host

The TCA cycle of the host in TB sputa was similar to the pattern previously described in MI inflammatory macrophages, broken after citrate and resulted in increased production of itaconate. The ACOI enzyme that converts citrate to cis-aconitate and isocitrate was significantly downregulated, while IRGI that mediates conversion to itaconate was significantly induced in TB sputa.
