# Supplementary material for: Transcriptomic Characterization of Tuberculous Sputum Reveals a Host Warburg Effect and Microbial Cholesterol Catabolism
Source: mBio. 2021 Dec 7;12(6):e01766-21. doi: 10.1128/mBio.01766-21 (PMC8649757; doi:10.1128/mBio.01766-21)
Supplement: FIG S1 [file mbio.01766-21-sf001.pdf]

SI Figure S1

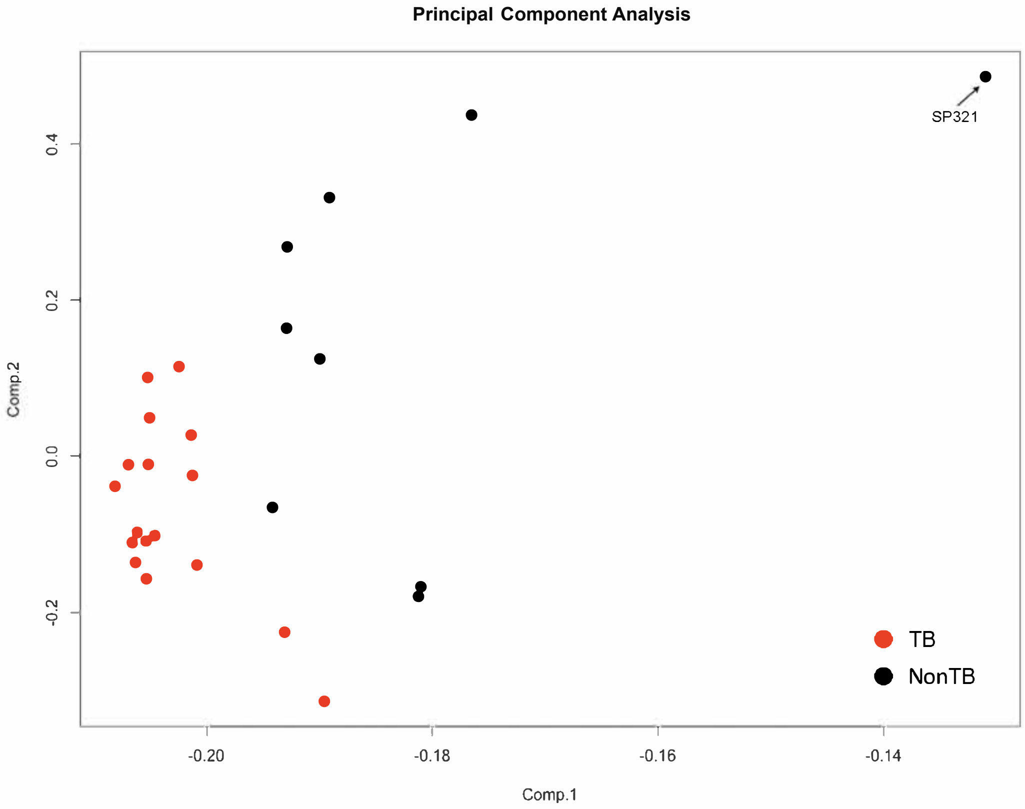

Supplemental Data Figure S1. Principal component analysis of host transcript profiles

The host transcriptomes of sputum samples were analyzed by principal component analysis. A complete segregation of the TB (red) from the non-TB (black) samples was observed. One of the non-TB sputa (SP321) was an outlier with differential clustering pattern and was excluded from downstream analysis of the host gene expression.
